# Mental health problems of asymptomatic or mildly symptomatic COVID-19 patients in hospitel in Thailand: A cross-sectional study

**DOI:** 10.12688/f1000research.125998.2

**Published:** 2022-10-07

**Authors:** Nitchawan Kerdcharoen, Pantri Kirdchok, Chayut Wonglertwisawakorn, Yingrat Naviganuntana, Nongnuch Polruamngern, Chotiman Chinvararak

**Affiliations:** 1 Department of Psychiatry, Faculty of Medicine Vajira Hospital, Navamindradhiraj University, Bangkok, 10300, Thailand

**Keywords:** Mental health problems; COVID-19; Hospitel; Thailand

## Abstract

Background

There is evidence that patients with COVID-19 have a higher prevalence of mental health problems than the normal population. This study aimed to investigate the prevalence of mental health problems and their associated factors in patients with asymptomatic or mildly symptomatic in the hospitel in Thailand.

Methods

Mental health problems were evaluated using the Depression, Anxiety, and Stress Scale - 21 items, and Patient Health Questionnaire-9. The prevalence of mental health problems was presented by frequency and percentage. McNemar's test was used to compare the prevalence of mental health problems between day 1 and day 7. Binary logistic regression was used to identify potential predictors of mental health problems.

Results

A total of 186 participants (68.3% female; mean age = 37.21 years (SD 13.66) were recruited. The depression, anxiety, and stress rate on day 1 of admission was 26.9%, 32.3% and 25.8%, respectively. Having mild COVID-19 symptoms was a significantly associated factor with anxiety (OR=2.69, 95%CI: 1.05-6.89) and stress (OR=4.53, 95%CI: 1.32-15.55).

Conclusions

There was a high rate of mental health problems in COVID-19 patients. Detecting and managing mental health problems should be considered standard care for COVID-19 patients.

## Introduction

Since the World Health Organization announced the emergency statement regarding the pandemic of novel coronavirus disease 2019 (COVID-19) on 30 January 2020, Thailand, like other countries, has to face the rise of new cases of COVID-19. The number of positive COVID-19 cases exceeded the health system's capacity. However, at the beginning of the pandemic, Thailand did not have a home isolation policy and stated that all patients would be under the medical team's care. Therefore, the Thai government set up the “hospitel”, a new type of health care facility. ^1^

The term “hospitel” is the compound noun from the “hospital” and “hotel”. It is a new type of health care facility specialised for COVID-19 patients who are asymptomatic or have only mild symptoms. A hospitel is organised and run by the hotel and medical staffs from an affiliated public hospital. The team includes general practitioners, nurses and paramedics. Patients in hospitel are monitored regularly and are quarantined for seven days or until they have negative COVID-19 test results. ^1^ Hence, hospitels also aim to prevent the spread of COVID-19 in the community. ^1^

Although patients are regularly monitored for physical complications, mental health problems, especially stress adjustment, may be under-recognised. The meta-analysis study by Liu *et al.*, 2021 found that anxiety symptoms and depression rates in COVID-19 patients were 32% and 27.6%, respectively. ^2^ Moreover, insomnia was found to have a prevalence of 30.30%. While the study in Thailand by Lerthattasilp *et al.*, 2020 showed that the prevalence of depression was found to be 22.5%, whilst the anxiety rate was 30%, and the stress rate was 20%. ^3^ The study by Lerthattasilp *et al.* was conducted in a field hospital that has a similar concept of caring to “hospitel”. ^3^ This data demonstrated that patients with COVID-19 are more likely to suffer mental health problems than the normal population. In addition, early studies revealed that the female gender, physical symptoms related to COVID-19, duration of hospitalisation, and a history of psychiatric disorders were associated factors to mental health problems. ^3^^–^^7^

The objective of the present study was to investigate the prevalence of mental health problems, including depression, anxiety and stress, as well as their associated factors in patients with asymptomatic or mildly symptomatic in the hospitel in Thailand, which is under the supervision of the Faculty of Medicine Vajira Hospital. The Faculty of Medicine Vajira Hospital is responsible for caring for COVID-19 patients from the Thonburi district in Bangkok. We hypothesise that the prevalence of mental health problems is likely to be high on day 1 at admission and will decline after 7 days. However, despite to the new outbreak of COVID-19 globally, there are still limited studies on mental health problems. Recognising this concern is essential to the Thai public health sector in order to implement appropriate measures to tackle mental health problems related to COVID-19 infection.

## Methods

### Ethics and consent

We obtained approval from the Ethical Committee of the Institutional Review Board of the Faculty of Medicine Vajira Hospital on July 2 ^nd^, 2021 (COA no. 120/64E). Before starting the survey, all participants were informed of the study's objectives, method, and provided written informed consent.

### Study design, setting and participants

We employed a cross-sectional descriptive study based on STROBE guidelines. ^8^ The sample size was calculated following the Cochrane formula. ^9^ As the number of COVID-19 patients (N) admitted to the hospitel between July and September 2021 was 250, the sample size was estimated by p = 0.198 according to the study by Jeong *et al.*, 2019. ^7^ Using alpha at 0.05 and error (d) at 0.05, the required sample size was 124. 186 asymptomatic or mildly symptomatic COVID-19 patients, according to COVID-19 treatment guidelines by the National Institutes of Health (NIH), aged 18 years and older were recruited by purposive sampling in-person when the participant was initially admitted to the hospitel under the supervision of the Faculty of Medicine Vajira Hospital from July to September 2021. Patients who could not use the internet were excluded from this study.

### Data collection

The study instruments consisted of four questionnaires: 1) demographic characteristics including sex, age, education level, employment status, financial status, and living status; 2) clinical characteristics including severity of COVID-19, duration of COVID-19 infection, duration admitted in the hospitel, referring status, admission status, history of medical and mental disorders and perceived psychological support while in the hospitel, which is a close-ended question (yes or no); 3) the Depression, Anxiety, and Stress Scale - 21 items (DASS-21); and 4) Patient Health Questionnaire-9 (PHQ-9) would performed if participants had moderate to severe severity from DASS-21 score in any domains. In addition, we collected participants' data on day 1 and day 7 of admission by Google form.

DASS-21 consists of three domains; each domain comprises seven items, and the depression, anxiety, and stress scores are calculated by summing. Then, the severity of each part is categorised into normal, mild, moderate, severe and extremely severe. The Cronbach's alpha coefficient of the DASS-21 Thai version is 0.75 reflecting good internal consistency. ^10^^,^^11^

PHQ-9 Thai version has a total of 9 depressive questions. The total score of PHQ-9 is classified into normal (0–6), mild (7–12), moderate (13–18), and severe (≥19). The sensitivity and specificity of PHQ-9 are 84% and 77%, respectively, to detect depression. ^12^^,^^13^

### Statistical analyses

Data were analysed using SPSS software (version 28.0; IBM, Chicago, IL, USA). The prevalence of mental health problems was presented by frequency and percentage. McNemar's test was used to compare the prevalence of mental health problems between day 1 and day 7. In addition, binary logistic regression (odds ratio [OR] and 95% confidence interval [CI]) was used to identify potential predictors of depression, anxiety and stress. P<0.05 was considered statistically significant.

## Results

Of 186 participants recruited in this study, they had a mean age of 37.21 years old (SD 13.66). The majority of participants were female (68.3%), single (54.8%), had an undergraduate degree (44.1%), employed (49.5%), and were living with family (59.7%) ( Table 1).

**Table 1.** Demographics of patients (N=186).

Variables	N	(%)
Age (years), Mean±SD	37.21±13.66	
(Min-Max)	(18-75)	
Sex		
Male	59	(31.7)
Female	127	(68.3)
Race: Thai	184	(98.9)
Marital status		
Single	102	(54.8)
Married	69	(37.1)
Divorced or Separated	11	(5.9)
Widow	4	(2.2)
Educational level		
Lower than primary school	5	(2.7)
Primary school	25	(13.4)
Junior high school	44	(23.7)
Senior high school	15	(8.1)
Undergraduate university	82	(44.1)
Postgraduate university	15	(8.1)
Occupation		
None	33	(17.7)
Government official	45	(24.2)
Self-employed	16	(8.6)
Employee	92	(49.5)
Income per month (Thai baht)		
0-5,000	23	(12.4)
5,001-10,000	28	(15.1)
10,001-15,000	37	(19.9)
15,001-20,000	32	(17.2)
20,001-25,000	12	(6.5)
>25,000	54	(29.0)
Living status		
Living alone	23	(12.4)
Living with friends	13	(7.0)
Living with a partner	39	(21.0)
Living with family	111	(59.7)
Family history of Mental disorder	9	(4.8)

Table 2 demonstrates the clinical characteristics of the patients. Approximately 16% of participants had at least one underlying medical illness. Only 1.1% of participants had an underlying mental disorder. In addition, around 80% of participants had mild COVID-19 symptoms, and the symptoms lasted at least 7 days. The median duration of hospitel admission was 12 days (IQR 10-13). Most participants were admitted alone (86.6%), and eventually, they could be discharged from the hospitel after 7 days of admission. Interestingly, around 90% of participants perceived that they were provided psychological support while in the hospitel.

**Table 2.** Clinical characteristics of patients (N=186).

Variables	N	(%)
Underlying medical disease	30	(16.1)
Diabetes mellitus	6	(3.2)
Hyperlipidaemia	10	(5.4)
Hypertension	20	(10.8)
Others	5	(2.7)
Underlying mental disorder	2	(1.1)
Depression	1	(0.5)
Anxiety	1	(0.5)
COVID-19 symptom		
Asymptomatic	35	(18.8)
Mild	151	(81.2)
Duration with COVID-19		
Asymptomatic	22	(11.8)
<7 days	35	(18.8)
≥7 days	129	(69.4)
Duration of hospitalisation (days)		
Median (IQR)	0	(0-0)
(Min-Max)	(0-12)	
Duration of hospitel admission (days)		
Median (IQR)	12	(10-13)
(Min-Max)	(2-14)	
Admission status		
Alone	161	(86.6)
With family	25	(13.4)
Family members diagnosed COVID-19		
Median (IQR)	1	(0-3)
(Min-Max)	(0-11)	
Referring status		
Discharge	164	(88.2)
Refer from hospital	19	(10.2)
Refer to hospital	3	(1.6)
Perceived psychological support		
No	18	(9.7)
Yes	168	(90.3)

**Figure 1.**
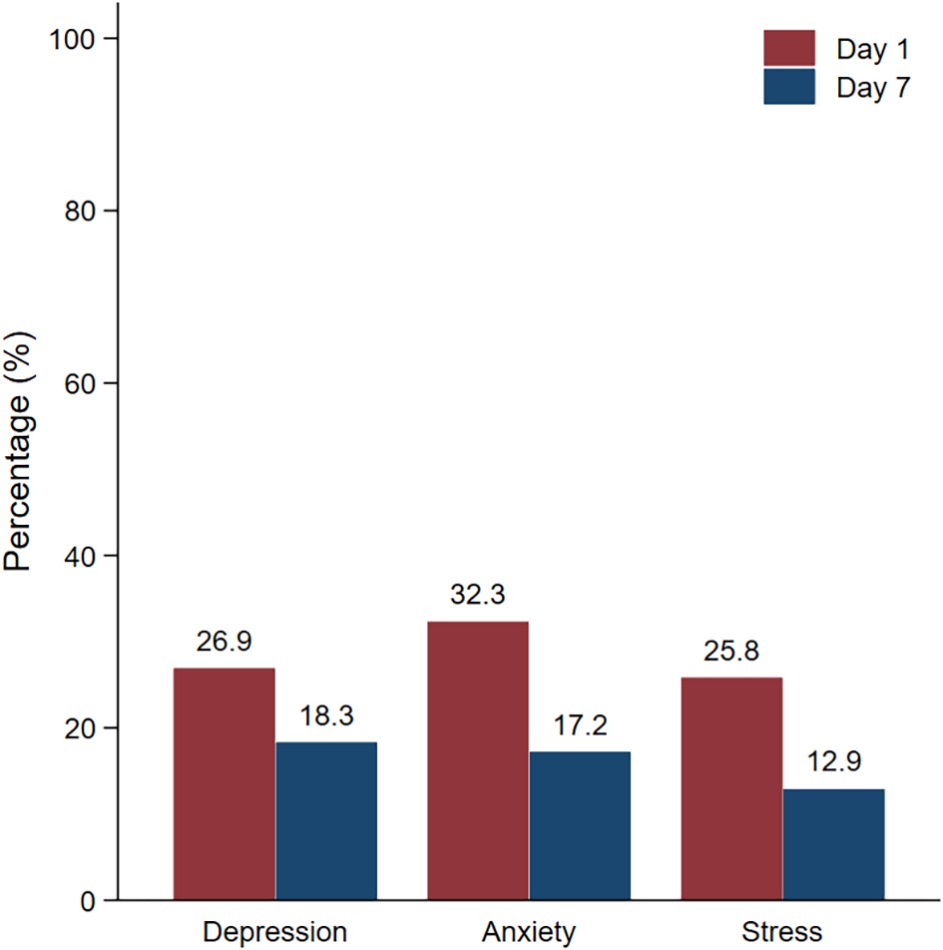
Prevalence of mental health problems on day 1 and day 7.

Regarding the prevalence of mental health problems ( Table 3), the depression, anxiety, and stress rates were 26.9%, 32.3% and 25.8%, respectively, on day 1 of hospitel admission. The most common level of depression measured by PHQ-9 was mild severity. However, after 7 days of admission, the depression, anxiety, and stress rates decreased to 18.3%, 17.2% and 12.9%, respectively. This difference in the proportion of mental health problems between day 1 and day 7 was statistically significant (P<0.05) ( Figure 1).

**Table 3.** Prevalence of mental health problems on day 1 and day 7 (N = 186).

Mental health problems	Day 1		Day 7		P-value ^a^
	N	(%)	N	(%)	
Depression assessed by DASS-21					
Normal	136	(73.1)	152	(81.7)	0.014 *
Mild to severe	50	(26.9)	34	(18.3)	
Anxiety					
Normal	126	(67.7)	154	(82.8)	<0.001 **
Mild to severe	60	(32.3)	32	(17.2)	
Stress					
Normal	138	(74.2)	162	(87.1)	<0.001 **
Mild to severe	48	(25.8)	24	(12.9)	
Depression assessed by PHQ-9, (n = 31)					
Normal	15	(48.4)	-	-	
Mild	12	(38.7)	-	-	
Moderate	3	(9.7)	-	-	
Severe	1	(3.2)	-	-	

^a^ McNemar's test.

* P<0.05.

** P<0.01.

The results of binary logistic regression analysis revealed that having mild COVID-19 symptoms was a significantly associated factor with anxiety (OR=2.69, 95%CI: 1.05-6.89) and stress (OR=4.53, 95%CI: 1.32-15.55). In contrast, other factors were not associated with depression, anxiety and stress (P>0.05) ( Table 4).

**Table 4.** Factors associated with mental health problems analysed by binary logistic regression.

Factors	Depression			Anxiety			Stress		
	OR	95%CI	P-value	OR	95%CI	P-value	OR	95%CI	P-value
Age (years)	1.00	(0.98-1.02)	0.928	1.00	(0.98-1.02)	0.923	0.98	(0.95-1.00)	0.081
Sex									
Male	1.15	(0.58-2.30)	0.686	1.55	(0.81-2.97)	0.183	1.61	(0.81-3.19)	0.176
Female	1.00	Reference		1.00	Reference		1.00	Reference	
Marital status									
Single	1.00	Reference		1.00	Reference		1.00	Reference	
Married	1.26	(0.63-2.50)	0.515	1.15	(0.60-2.20)	0.685	0.51	(0.24-1.07)	0.076
Widow/Divorced/Separated	1.54	(0.48-4.93)	0.467	1.53	(0.50-4.66)	0.457	1.60	(0.52-4.89)	0.410
Education level									
Primary school or lower	1.37	(0.57-3.30)	0.489	1.02	(0.43-2.42)	0.972	0.94	(0.38-2.37)	0.900
High school	0.85	(0.40-1.80)	0.670	0.89	(0.44-1.79)	0.747	0.73	(0.34-1.57)	0.422
Undergraduate university or higher	1.00	Reference		1.00	Reference		1.00	Reference	
Occupation									
Government official	1.00	Reference		1.00	Reference		1.00	Reference	
Self-employed/Employee	1.86	(0.78-4.45)	0.162	1.75	(0.80-3.83)	0.164	1.41	(0.62-3.19)	0.411
None	2.31	(0.81-6.63)	0.119	1.34	(0.49-3.68)	0.565	0.94	(0.32-2.81)	0.915
Income (baht)									
0-15,000	1.24	(0.56-2.75)	0.600	1.17	(0.56-2.43)	0.679	0.90	(0.41-1.96)	0.782
15,001-25,000	1.81	(0.74-4.43)	0.193	1.23	(0.52-2.89)	0.637	1.20	(0.49-2.91)	0.690
>25,000	1.00	Reference		1.00	Reference		1.00	Reference	
Living status									
Living alone	0.57	(0.18-1.81)	0.338	0.49	(0.17-1.43)	0.193	0.60	(0.19-1.90)	0.380
Living with friends	1.69	(0.51-5.57)	0.390	1.52	(0.48-4.84)	0.477	1.77	(0.54-5.84)	0.350
Living with a partner	1.06	(0.47-2.39)	0.887	0.53	(0.23-1.23)	0.141	0.98	(0.42-2.25)	0.953
Living with family	1.00	Reference		1.00	Reference		1.00	Reference	
Underlying medical disease	0.64	(0.24-1.66)	0.356	0.47	(0.18-1.23)	0.123	0.53	(0.19-1.46)	0.218
Underlying mental disorder	-	-	NA	-	-	NA	2.92	(0.18-47.53)	0.453
Family history of mental	2.28	(0.59-8.85)	0.234	2.77	(0.72-10.73)	0.140	2.42	(0.62-9.41)	0.203
COVID-19 symptom									
Asymptomatic	1.00	Reference		1.00	Reference		1.00	Reference	
Mild	2.55	(0.93-6.99)	0.069	2.69	(1.05-6.89)	0.039 *	4.53	(1.32-15.55)	0.03 *
Admission status									
Alone	1.00	Reference		1.00	Reference		1.00	Reference	
With family	1.65	(0.68-4.01)	0.272	1.48	(0.62-3.52)	0.375	0.90	(0.34-2.39)	0.824
Family members diagnosed with COVID-19	1.04	(0.90-1.20)	0.616	1.03	(0.89-1.18)	0.692	0.94	(0.80-1.11)	0.481
Perceived psychological support									
No	2.40	(0.89-6.48)	0.084	1.38	(0.51-3.76)	0.528	1.12	(0.38-3.32)	0.841
Yes	1.00	Reference		1.00	Reference		1.00	Reference	

Abbreviations: OR, Odds Ratio; CI, confident interval.

* P<0.05.

## Discussion

To the best of our knowledge, this is the first study to explore the prevalence of mental health problems among patients with COVID-19 in the hospitel in Thailand. The prevalence of depression was 26.9%, anxiety was 32.3%, and stress was 25.8% in patients with asymptomatic or mild COVID-19 symptoms at day 1 of their stay at the hospitel under the Faculty of Medicine Vajira Hospital supervision. Compared to the meta-analysis study from multinational countries, including China, the United States, Japan, India, and Turkey, the depression and anxiety rates had a similar trend (27.6 % vs 26.9 for depression and 32.6% vs 32.3% for anxiety). ^2^ On the contrary, the stress rate in this study was relatively lower than in the study at the Thammasat University field hospital (30% vs 25.8%). ^3^

Although we also used the DASS-21, the same questionnaire, the prevalence of stress in this study might have been lower since the study at the Thammasat University field hospital had more moderate to severe COVID-19 cases. ^3^ In addition, the context of hospitels and field hospitals were different in many ways; for example, the privacy and facility of hospitels might be better than field hospitals.

The mental health problems rate declined significantly on day 7 of admission (P<0.05). The potential explanation may be that most patients can adjust to acute stress over time and with perceived psychological support. ^14^^,^^15^ Moreover, the medical team at the hospitel always provide basic psychoeducation via a leaflet and video clip about coping with stress. ^16^ The high-risk cases of mental disorders would then be referred to psychologists or psychiatrists.

In this study, mild COVID-19 symptoms was the only factor associated with anxiety and stress. This could be a helpful predictor of psychological screening problems in patients admitted to hospitel. However, unlike prior studies, we could not find the association between the female gender, duration of hospitalisation, and a history of psychiatric disorders and mental health problems. This could be because there were few patients with psychiatric disorders in this study. Additionally, we did not collect data on the detail of the physical symptoms of COVID-19.

We are aware of some limitations of the present study. First, we can only indicate associated factors, not causal relationships, due to the descriptive design. Secondly, we included only asymptomatic and mild symptoms, which may not represent all COVID-19 patients. Finally, the mental health problems in this study were assessed by online self-reporting questionnaires, which could demonstrate only symptoms, not disorders and patients who could not use the internet were excluded. Thus, patients with high-risk mental disorders should be further evaluated by psychiatrists or clinical psychologists.

Future research should investigate the prevalence of posttraumatic stress disorder (PTSD), which could be occurred following COVID-19 as a traumatic stressor. ^17^ In addition, psychological intervention to prevent stress-related illnesses or psychological distress ^18^ should be performed.

## Conclusions

The prevalence of mental health problems in COVID-19 patients was common, especially on the first day of admission. However, it declined on the 7th day after admission. In addition, having mild symptomatic COVID-19 infection was an associated factor with anxiety and stress. Therefore, detecting and managing mental health problems should be considered standard care for COVID-19 patients.

## Data availability

### Underlying data

figshare: Mental health problems of asymptomatic or mildly symptomatic COVID-19 patients in hospitel in Thailand: A Cross-Sectional Study, https://doi.org/10.6084/m9.figshare.21108790.v1. ^19^

This project contains the following extended data:

• Hospitel Data.sav (anonymised responses in spss)

### Extended data

figshare: Mental health problems of asymptomatic or mildly symptomatic COVID-19 patients in hospitel in Thailand: A Cross-Sectional Study, https://doi.org/10.6084/m9.figshare.21108790.v1. ^19^

This project contains the following extended data:

• Demographic data record-Hospitel.docx (blank English copy of the demographic and clinical characteristics questionnaire used in this study)

Data are available under the terms of the Creative Commons Zero “No rights reserved” data waiver (CC0 1.0 Public domain dedication).
