# Supplementary material for: Mental health problems of asymptomatic or mildly symptomatic COVID-19 patients in hospitel in Thailand: A cross-sectional study
Source: F1000Res. 2025 Apr 1;11:1089. Originally published 2022 Sep 22. [Version 4] doi: 10.12688/f1000research.125998.4 (PMC9845800; doi:10.12688/f1000research.125998.4)
Supplement: Peer review history

# Reviewer report: Reviewer response for version 2

**DOI:** 10.5256/f1000research.139474.r159686

**Published:** 2023-01-17

**Authors:** Dr. Richard Mottershead

**Affiliations:** 1 Ras Al Khaimah College of Nursing, RAK Medical and Health Sciences University, Ras Al Khaimah, United Arab Emirates

Thank you for allowing me to review your relevant and topical paper. The paper provides a useful insight into the impact of Covid-19 not only on the physical aspect but the mental health implications of the virus and wider pandemic fall-out. 

 The authors introduce a rich data stream that provides a useful insight into care in Thailand which can be then examined by researchers globally based on the methods and methodology set down within this article.  

 The recommendations of ensuring mental health care for those having been diagnosed with Covid-19 is an intervention which would provide holistic care for our communities and would benefit our wider national healthcare strategies.

Is the work clearly and accurately presented and does it cite the current literature?

Yes

If applicable, is the statistical analysis and its interpretation appropriate?

Yes

Are all the source data underlying the results available to ensure full reproducibility?

Yes

Is the study design appropriate and is the work technically sound?

Yes

Are the conclusions drawn adequately supported by the results?

Yes

Are sufficient details of methods and analysis provided to allow replication by others?

Yes

Reviewer Expertise:

I am a researcher who is actively involved in health and social care and applied research for disenfranchised groups and areas of need within our society.

I confirm that I have read this submission and believe that I have an appropriate level of expertise to confirm that it is of an acceptable scientific standard.

# Reviewer report: Reviewer response for version 2

**DOI:** 10.5256/f1000research.139474.r152674

**Published:** 2022-10-10

**Authors:** Sorawit Wainipitapong

**Affiliations:** 1 Department of Psychiatry, Faculty of Medicine, Chulalongkorn University, Bangkok, Thailand

The authors responded to all suggestions with a satisfactory revision. Thank you for revising according to my comments and I have no further queries or suggestions.

Is the work clearly and accurately presented and does it cite the current literature?

Yes

If applicable, is the statistical analysis and its interpretation appropriate?

Partly

Are all the source data underlying the results available to ensure full reproducibility?

Yes

Is the study design appropriate and is the work technically sound?

Yes

Are the conclusions drawn adequately supported by the results?

Yes

Are sufficient details of methods and analysis provided to allow replication by others?

Yes

Reviewer Expertise:

Psychiatry

I confirm that I have read this submission and believe that I have an appropriate level of expertise to confirm that it is of an acceptable scientific standard.

# Reviewer report: Reviewer response for version 1

**DOI:** 10.5256/f1000research.138366.r151293

**Published:** 2022-09-26

**Authors:** Sorawit Wainipitapong

**Affiliations:** 1 Department of Psychiatry, Faculty of Medicine, Chulalongkorn University, Bangkok, Thailand

Thank you for the opportunity to review this article. The report was well written and organized.

 Hereby, please find my suggestion for this work to become better suitable for indexing:

1. Some general profiles of study location, Vajira Hospital, should be stated. For example, is it located in the metropolitan, urbanized, or regional area? Readers would gain benefit for generalizability regarding mental health-related profiles and conditions.
2. Are previous mental disorders enrolled in the exclusion criteria?
3. How did the authors measure ‘perceived psychological support while in the hospitel’? Please clarify.
4. Please kindly check the psychometric properties of PHQ-9 Thai version. Because the categorized severity of depression and the cut-off score of two cited references are not similar, the authors are suggested to carefully use the interpretation of this screening tool and specify the cut-off score used in the study.
5. Table 1 – Does ‘income’ refer to ‘income per month’?
6. Table 2 – Should ‘asymptomatic’ in the ‘Duration with COVID-19’ section be placed in the proper position?
7. Figure 1 – P-value could also be shown in the figure for it was mentioned in the text.
8. Table 4 – Please check the indent used in the table (i.e. Widow/Divorced/Separated and Educational level).
9. Table 4 – Please check the spelling ‘P<0.0.’ in the end of the table.
10. Compared to the finding from another Thai field hospital, the authors might provide additional discussions regarding the location or uniqueness of the hospital besides the severity of COVID-19.

Congratulations to all authors. They have done great work and I enjoyed reading this manuscript a lot.

Is the work clearly and accurately presented and does it cite the current literature?

Yes

If applicable, is the statistical analysis and its interpretation appropriate?

Partly

Are all the source data underlying the results available to ensure full reproducibility?

Yes

Is the study design appropriate and is the work technically sound?

Yes

Are the conclusions drawn adequately supported by the results?

Yes

Are sufficient details of methods and analysis provided to allow replication by others?

Yes

Reviewer Expertise:

Psychiatry

I confirm that I have read this submission and believe that I have an appropriate level of expertise to confirm that it is of an acceptable scientific standard, however I have significant reservations, as outlined above.

# Response

**Published:** 2022-09-26

**Authors:** Chotiman Chinvararak

**Affiliations:** Faculty of Medicine Vajira Hospital, Navamindradhiraj University, Thailand

Thank you very much for your review. We will try to address all your comments and improve them in version 2 of this paper.

 1. Vajira hospital is located in Bangkok and is responsible for caring for COVID-19 patients, especially those from the Thonburi district.

 2.  We did not exclude participants with previous mental disorders. There were 2 participants with preexisting mental disorders (shown in Table 2).

 3. We used close-ended question (yes or no) to measure perceived psychological support while in the hospitel. We will update this information in version 2 of this paper.

 4. We used the classification of depression (PHQ-9 Thai version) following reference number 13.

 5. Yes, it refers to income per month. We will add this detail in version 2 of this paper.

 6. We will change the position of "asymptomatic" in version 2 of this paper.

 7. We will demonstrate P-value in Figure 1.

 8. We will check the indent used in Table 1.

 9. We will correct this mistake. The phrase is P<0.05.

 10. We will discuss this issue (location of the hospitel) in version 2 of this paper.
